# Adenovirus Disease and Ocular Symptoms in Children: Diagnosis and Prognostic Considerations

**DOI:** 10.1155/crdi/2621782

**Published:** 2025-08-06

**Authors:** Jannatul Fardous, Arpita Goutam, Tajrin Rahman, Zahid Hasan Khan, Sabrina Nahin, Sumaiya Hussain, Mohammad Safiul Bashar Khan, Mohammad Delwer Hossain Hawlader, Mohammad Ashraful Amin

**Affiliations:** ^1^Department of Paediatric Nephrology, National Institute of Kidney Diseases & Urology (NIKDU), Dhaka 1207, Bangladesh; ^2^Department of Public Health, North South University, Dhaka 1229, Bangladesh; ^3^Department of Public Health, Public Health Promotion and Development Society (PPDS), Dhaka 1205, Bangladesh; ^4^Department of Enteric and Respiratory Infections, International Centre for Diarrhoeal Disease Research, Bangladesh (icddr,b), Dhaka 1212, Bangladesh; ^5^Department of Physiology, Green Life Medical College Hospital, Dhaka 1205, Bangladesh; ^6^Department of Ophthalmology, Bangladesh College of Physicians & Surgeons (BCPS), Dhaka 1212, Bangladesh; ^7^Department of Medicine, Prince Mohammad Bin Abdulaziz National Guard Hospital, Modinah, Saudi Arabia; ^8^Department of Clinical Trials, London School of Hygiene and Tropical Medicine, London, UK

## Abstract

Adenoviral infections significantly impact pediatric health, manifesting as respiratory, gastrointestinal, and ocular disorders. We report a 14-year-old male with adenoviral pharyngoconjunctival fever (PCF) complicated by subconjunctival hemorrhage and enlarged adenoids. The patient presented with high-grade fever, sore throat, bilateral conjunctivitis, and gastrointestinal symptoms, including vomiting and diarrhea. Initial laboratory findings suggested septicemia; however, an extended respiratory panel confirmed adenoviral infection. Notably, the patient also had left-sided undescended testes and scoliosis, raising questions about potential associations. This case underscores the complexity of adenoviral infections in children, highlighting the interplay of respiratory, gastrointestinal, and ocular symptoms. The presence of additional conditions warrants further investigation into possible correlations with adenoviral infection. Comprehensive evaluation is crucial for accurate diagnosis and management of adenoviral infections in pediatric patients, and future research should explore long-term implications and associations with cryptorchidism and scoliosis.

## 1. Introduction

Adenoviral infections are a significant public health concern, particularly among pediatric populations. By the age of ten, most children will have experienced at least one adenovirus infection, with the highest incidence occurring in infants and children aged 6 months to 5 years [1]. Adenoviruses are capable of causing a broad spectrum of illnesses, affecting the respiratory, gastrointestinal, urinary, ocular, and hepatic systems. Ocular manifestations of adenoviral infection are categorized into three primary syndromes: simple follicular conjunctivitis, pharyngoconjunctival fever (PCF), and epidemic keratoconjunctivitis (EKC) [2]. Notably, serotype D has been linked to EKC and ocular infections [3].

The incubation period for adenoviral infections ranges from 2 to 14 days, with transmission occurring via respiratory secretions, direct contact, aerosolized particles, contaminated surfaces, and the fecal-oral route [4]. Acute follicular conjunctivitis, the most prevalent adenoviral ocular infection, typically presents with unilateral conjunctival follicular lesions [1]. PCF, primarily associated with adenovirus types 3 and 7, is another notable manifestation [5]. Adenoviral conjunctivitis accounts for 15 to 70% of all infectious conjunctivitis cases worldwide, with a higher prevalence of 65–90% among viral conjunctivitis cases. The condition manifests in four recognized clinical phenotypes: EKC, PCF, acute nonspecific follicular conjunctivitis, and chronic keratoconjunctivitis. Among these, EKC, linked to serotypes 8 and 19, represents the most severe form, often resulting in significant long-term ocular morbidity [6]. Although EKC is more commonly observed in adults, cases in children under 2 years old have been documented [1].

The diagnosis of adenoviral conjunctivitis can be complex due to symptom overlap with other ocular infections. Traditional diagnostic approaches, including viral cell culture and polymerase chain reaction (PCR), are gradually being supplanted by advanced techniques such as whole genome sequencing, which offer enhanced diagnostic accuracy. This case report deals with a 14-year-old male who was experiencing adenoviral PCF, which is further worsened by enlarged adenoids and subconjunctival hemorrhaging.

## 2. Case History

A 14-year-old boy presented with high-grade intermittent fever, chills, and rigor lasting four days. The fever was persistent despite antipyretics. The patient also reported abdominal pain, sore throat, redness of both eyes, and a watery discharge. He experienced several episodes of vomiting with undigested food particles and passed loose watery stools twice prior to admission.

The child was admitted to the hospital for 8 days. His medical history includes a tonsillectomy performed 4 years ago, and he is up-to-date with immunizations, including those against COVID-19. Family history reveals bronchial asthma, but there was no known contact with tuberculosis (TB) or documented weight loss. The patient's birth history indicates he was delivered by lower uterine cesarean section (LUCS) at term with a birth weight of 4.5 kg from a nondiabetic mother, with no perinatal complications. Developmental milestones were appropriate for his age.

**Figure 1:**
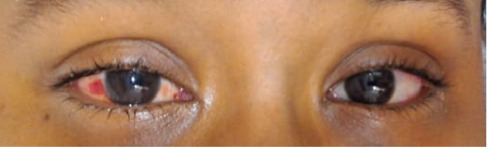
Bilateral conjunctival congestion and watery discharge with subconjunctival hemorrhage observed due to adenovirus.

On examination, the patient appeared ill but was conscious, cooperative, and febrile with a temperature of 103°F. Vital signs were as follows: pulse 98/min, blood pressure 120/70 mmHg, respiratory rate 24/min with transmitted sounds, and oxygen saturation maintained in room air. He was diagnosed with postural scoliosis and left-sided undescended testes. The ophthalmologist saw and noted bilateral conjunctival congestion and watery discharge was noted, with subconjunctival hemorrhage observed (Figure 1). The cornea was clear, pupils were reactive, visual acuity was reasonable, and mild lid swelling was present without conjunctival membrane. Postauricular lymph nodes were palpable. The throat was congested, but there were no signs of meningeal irritation. The BCG vaccination mark was evident. The relevant investigation that was done revealed WBC and CRP were high (Table 1). The extended respiratory panel detected adenovirus. The diagnostic evaluation includes PCR testing for adenovirus, serological assays, and conjunctival swabs, which aid in confirming the viral etiology. Anterior segment photography and ocular coherence tomography (OCT) were assessing ocular involvement and monitoring disease progression. Quantitative RT-PCR for urinary CMV was negative, and both throat and urine cultures, as well as blood cultures, showed no growth.

Subsequent examination by a general physician revealed no abnormalities on abdominal or other systemic examination. Treatment was initiated with empirical intravenous antibiotics (carbapenem) and antivirals (acyclovir) to cover potential secondary bacterial infection and viral pathogens while awaiting confirmatory results. Upon identification of adenovirus and exclusion of bacterial co-infection, the focus shifted to supportive care. Symptom-specific treatments included IV fluids to manage dehydration, ondansetron and sucralfate for gastrointestinal symptoms, and eye drops (moxifloxacin, ciprofloxacin, and carboxymethylcellulose) for ocular relief. Additionally, antihistamines (levocetirizine) and leukotriene receptor antagonists (montelukast) were used to alleviate respiratory and allergic symptoms. The management approach was multidisciplinary, involving pediatricians, ophthalmologists, and infectious disease specialists, and was guided by clinical progression and lab findings. This comprehensive strategy ensured appropriate care tailored to the multiorgan involvement of adenoviral infection (Table 2). Additionally, the physician emphasized the importance of infection control measures, such as hand hygiene and isolation protocols, to prevent nosocomial outbreaks and household transmission. Initially, septicemia was suspected, and the patient was treated with intravenous antibiotics and supportive measures. By the sixth day of hospitalization, the patient's condition showed marked improvement. The fever resolved completely, vomiting and diarrhea subsided, and his appetite began to return. Ocular symptoms, including redness and discharge, reduced significantly, and the subconjunctival hemorrhage started to regress. The lid swelling diminished, and there was no new ocular involvement. The patient was discharged on day eight in a stable condition, with instructions for continued home care and symptom monitoring. A follow-up visit was scheduled for 1-week postdischarge. During this outpatient visit, the patient remained symptom-free. There was complete resolution of conjunctival congestion and subconjunctival hemorrhage, and visual acuity had returned to baseline. Ophthalmologic reassessment confirmed a normal anterior segment with no signs of corneal involvement or residual inflammation. The patient was advised to follow up with pediatric surgery for evaluation of the left-sided undescended testes and referred to orthopedics for further assessment of scoliosis.

## 3. Discussion

This case report of a 14-year-old male with adenoviral PCF accompanied by subconjunctival hemorrhage, enlarged adenoid, left-sided undescended testis, and scoliosis opens a wider view on the multifaceted and rather complex clinical picture of adenoviral infection. The clinical presentation of high-grade intermittent fever, sore throat, bilateral conjunctival congestion, and watery discharge corresponds with the typical signs of PCF, which are fever, conjunctivitis, and pharyngitis [8, 9].

In addition to the classical symptoms of PCF, both postauricular lymph nodes were palpable in this case. Other studies have observed lymphadenopathy in regions such as preauricular or cervical submandibular areas, highlighting the variability in lymphatic involvement with this condition [9, 10]. Although subconjunctival hemorrhage is relatively rare in association with adenoviral infections, its occurrence may suggest a more severe inflammatory response or vascular compromise warranting further investigation into its prevalence and significance in pediatric patients [11, 12]. The presence of gastrointestinal symptoms, notably episodes of vomiting and watery diarrhea, adds another level of complexity to the case. Similar manifestations were documented in cases reported by Bye et al. and Gupta et al., which involved patients presenting fever, conjunctivitis, and gastrointestinal complaints [13, 14].

While the connection between undescended testis and adenoviral infection remains unexplored in the literature, this case highlights the potential for correlations that merit further investigation. Elevated fetal estradiol levels caused by viral infection during pregnancy may cause hypogonadotropic, hypogonadism, cryptorchidism, and retarded epididymal development [15]. Adenoviruses can infect various tissues, and vertical transmission during pregnancy has been reported [16]. Viral infections during pregnancy can lead to inflammatory responses, apoptosis (programmed cell death), or cytotoxic effects, all of which can have detrimental effects on developing tissues and organs. These disruptions can interfere with normal fetal development, potentially resulting in congenital abnormalities. Excessive inflammation during critical periods of organogenesis (the process of organ formation in the fetus) can lead to tissue damage, disrupted cell signaling, and abnormal development. This might or can lead to conditions such as cryptorchidism or skeletal development disturbances that may be linked to scoliosis. Another possible mechanism is the immunological response to adenoviral infection, which triggers systemic immune activation and elevated levels of proinflammatory cytokines like IL-6, IL-1β, and TNF-α. These cytokines can disrupt fetal tissue differentiation and organogenesis, potentially leading to congenital anomalies. Prolonged immune activation during critical developmental periods may alter normal growth patterns and structural formation [16–19]. However, in our patient, there is no documented history of previous adenoviral infection, and the current episode of adenoviral illness cannot be directly linked to undescended testes. Given the lack of prior medical records indicating adenoviral exposure during fetal development, it is more plausible that the undescended testes in this case represent a congenital anomaly unrelated to the current adenoviral infection.

Scoliosis, an idiopathic condition, is influenced by genetic, growth, and neurological factors, along with changes in bone mass density (BMD), tissue abnormalities, and imbalances in specific chemicals within the body [20]. Although a direct link between adenoviral infection and scoliosis is yet to be established, individuals with scoliosis may be more severely affected in case of viral respiratory tract infection including reduced vital capacity due to muscular weakness or spastic scoliosis [21]. The initial concern for septicemia, prompted by laboratory findings of neutrophilic leukocytosis and elevated C-reactive protein levels, underscored the potential severity of the illness. However, an extended respiratory panel detected adenovirus. While throat, blood, and urine cultures showed no growth, further reinforcing the viral nature of the infection. Given the self-limiting nature of most adenoviral infections, patient education and reassurance remain crucial components of management. Caregivers should be advised on the typical disease course, expected symptom duration, and the importance of follow-up in cases with persistent or worsening symptoms.

For severe ocular manifestations, such as persistent keratitis or corneal involvement, close ophthalmologic monitoring is recommended. Topical corticosteroids may be used cautiously under specialist supervision to minimize complications such as secondary glaucoma. In cases where significant ocular discomfort or photophobia persists, nonsteroidal anti-inflammatory drugs (NSAIDs) may be beneficial in conjunction with artificial tears. Furthermore, in pediatric patients at risk of prolonged viral shedding, especially those with underlying immunosuppression, adjunctive measures such as intravenous immunoglobulin (IVIG) have been explored in rare cases, although evidence remains limited. The limitations of this case include the lack of comprehensive periodic follow-up to assess long-term outcomes and the absence of investigation into potential correlations between adenoviral infection and undescended testis or scoliosis. Additionally, the variability in lymphatic involvement and the rarity of subconjunctival hemorrhage associated with adenoviral infections warrant further study to confirm prevalence and significance.

In conclusion, this case underscores the importance of a comprehensive approach to diagnosing and managing adenoviral infections in pediatric patients. The interplay of gastrointestinal, respiratory, and ocular symptoms necessitates a thorough evaluation to ensure accurate diagnosis and appropriate treatment [7].

## Figures and Tables

**Table 1:** Patient laboratory findings.

Name of the test	Finding
Complete blood count (CBC)	
Hemoglobin	10.7 g/dL
Total count of white blood cell (WBC)	15,000/cmm
Differential count: Neutrophil	80%
Lymphocyte	14%
Platelet count	300,000/cmm
C-reactive protein (CRP)	45.6 mg/L
Erythrocyte sedimentation rate (ESR)	59 mm
Peripheral blood film (PBF)	Microcytic hypochromic anemia with neutrophilic leukocytosis
S. Electrolytes:	
Serum sodium	133 mmol/L
Serum potassium	4 mmol/L
Serum chloride	98 mmol/L
TCO2	24 mmol/L
S. Creatinine	0.7 mmol/L
Mantoux test (MT)	Negative
Dengue NS1 ag and ICT	Negative
Cytomegalovirus (CMV) IgM:	Negative
Cytomegalovirus (CMV) IgG:	72.4 AU/mL (positive)
Serum glutamic pyruvic transaminase (SGPT)	59 U/L
SGOT/AST (aspartate amino transferase)	19 U/L
Lactate dehydrogenase (LDH)	214 U/L
Prothrombin time (PT)	13 s
Activated partial thromboplastin time (APTT)	33.7 s
International normalized ratio (INR)	1.06
D-dimer	0.36 mg/L
Fibrinogen degradation products (FDP)	4.70 μg/mL
Febrile ag: OX2	< 1:160
Troponin-I:	1.29 pg/mL
Urine routine examination (R/E):	
Sediment	Sediment 2+
Albumin	+
Pus cell	5–6/HPF

**Table 2:** Ongoing treatment of the patients during hospitalization.

1. Intravenous (IV) fluid (5% DNS)
2. Injection. carbapenem (1 gm 8 hourly)
3. Injection. Acyclovir (500 mg 8 hourly)
4. Tablet Pantoprazole
5. Injection. Ondansetron
6. Sucralfate
7. Tablet. Drotaverine
8. Mouthwash
9. Moxifloxacin eye drop
10. Ciprofloxacin eye drop
11. Carboxymethylcellulose sodium eye preparation
12. Tablet. Levocetrizine
13. Tablet. Montelukast

## Data Availability

The data that support the findings of this study are available from the corresponding author upon reasonable request.
